# Cleft Lip and/or Palate Is Associated with an Increased Prevalence of Mental Health and Behavioral Disorders

**DOI:** 10.1177/10556656251331329

**Published:** 2025-04-30

**Authors:** F. Jeffrey Lorenz, Cheng Ma, Aniruddha C. Parikh, Jessyka G. Lighthall

**Affiliations:** 1 Department of Otolaryngology—Head and Neck Surgery, 12311 Penn State Hershey Medical Center, Hershey, PA, USA

**Keywords:** cleft lip, cleft palate, social support, quality of life, psychosocial adjustment

## Abstract

**Objective:**

To evaluate the prevalence of mental health and behavioral disorders in individuals with cleft lip and/or palate (CL/P) versus the general population.

**Design:**

Retrospective cohort study. Cohorts of people with CL/P ≤ 18 and >18 years of age were compared to controls matched for age and sex.

**Setting:**

92 healthcare organizations.

**Patients, Participants:**

The TriNetX Research Network was queried for pediatric and adult participants using diagnosis and procedure codes from 2011 to 2021.

**Main Outcome Measure(s):**

Rates of mental health disorders and prescriptions for mental health medications and stimulants.

**Results:**

Pediatric (*n* = 45,341) and adult (*n* = 10,855) individuals with CL/P were significantly more likely than controls to have depressive episodes, anxiety disorders, attention-deficit hyperactivity disorders, and disruptive disorders, and be prescribed mental health medications and stimulants (all *P* < .001). Relative risks compared to controls for mental health diagnoses and medications ranged from 2.32 to 5.47 for pediatric participants and from 1.26 to 10.79 for adults. Pediatric participants with a cleft palate with or without a cleft lip and associated conductive hearing loss (CHL) or otitis media with effusion (OME) showed higher rates of disorders and prescriptions compared to those without CHL/OME (all *P* < .001).

**Conclusions:**

CL/P, especially when associated with OME/CHL, was associated with a significantly higher prevalence of mental health and behavioral disorders. These effects were present across age groups, underscoring the need for early intervention strategies and long-term mental health support for individuals with CL/P.

## Introduction

Cleft lip and/or palate (CL/P) is among the most common congenital anomalies, affecting approximately one in 700 live births worldwide and one in 1000 in the United States.^1^ These craniofacial conditions may occur as a component of genetic syndromes or in isolation,^2^ and result from incomplete fusion of the lip and palate during embryonic development.^3^ This leads to a spectrum of clinical manifestations that can detrimentally impact feeding, speech, hearing, dental development, and aesthetic appearance.^4^ Management of CL/P involves a multidisciplinary approach, integrating the expertise of otolaryngologists, plastic surgeons, dentists, orthodontists, geneticists, psychologists, speech-language pathologists, and audiologists to address the diverse needs of affected individuals.^5^

Beyond physical and functional challenges, individuals with CL/P often face significant psychosocial burdens, including heightened risks of psychological distress, stigmatization, social isolation, and low self-esteem.^6‐8^ Existing research suggests a potential association between the various challenges faced by individuals with CL/P and a higher prevalence of mental health disorders,^9‐13^ though direct causality has not been established. There is general agreement that individuals with CL/P are at increased risk for mental health disorders, and it has also been reported that those with cleft palate (CP) or cleft lip and palate (CLP) may experience the highest rates of psychiatric comorbidities, however this varies across studies.

Additionally, hearing loss, particularly conductive or mixed hearing loss (CHL), is one of the most common functional impairments in children with CL/P, affecting up to 80% of this group.^14,15^ This is primarily due to chronic otitis media with effusion (OME), which stems from Eustachian tube dysfunction.^16^ In other populations, hearing loss has been well-documented to contribute to social withdrawal and an increased risk of mental health disorders.^17,18^ This raises the hypothesis that youth with CL/P and associated hearing loss may face an even greater risk for psychological challenges.

The objective of this study was to determine the prevalence of mental health and behavioral disorders in pediatric and adult individuals with CL/P on a national scale with a large sample size. A secondary aim was to explore whether CHL/OME influence the rates of mental health conditions. Understanding these associations is crucial for developing targeted interventions and support systems for this vulnerable population.

## Methods

The data for this study was obtained from the TriNetX Research Network. TriNetX is a comprehensive database based on diagnosis (ICD-10) and procedure (CPT) codes extracted from the electronic medical records of 107 healthcare organizations (HCOs) across the U.S. and internationally, representing over 130 million patients.^19^ Of the 107 HCOs, 37 are international including 25 from Brazil, one from Colombia, one from Georgia, one from India, and nine from Taiwan. TriNetX is designed to facilitate clinical research by providing real-world data in aggregate and de-identified form, ensuring compliance with the Health Insurance Portability and Accountability Act (HIPAA). The Institutional Review Board ruled STUDY00018629 as exempt research according to institutional policies and relevant federal regulations. There were 92 out of 107 HCOs which contributed data to this study, comprising 51 academic and 41 private institutions from across the United States as well as a small percentage from outside the country. While it was possible to determine the total number of U.S. versus non-U.S. individuals, it is was not possible to determine the breakdown of the HCOs contributing data to the cohorts. Although the database does not include information on insurance coverage, it encompasses patients with a range of payment sources, as well as those without insurance.

The database was queried via ICD-10 codes to identify participants with a documented diagnosis of CL/P during 2011 through 2021. The codes used for defining the search criteria and conducting all analyses are detailed in Supplement 1. The decision was made to use ICD-10 codes instead of CPT codes to define the cohort, as requiring CPT codes for cleft surgeries would have been overly restrictive, potentially excluding older adolescents and adults who may have undergone surgery at different institutions and limiting the ability to study the development of mental health disorders across all age groups. Additionally, there are unique ICD-10 codes for cleft lip (CL), CP, and CLP. Since ICD-10 codes were not in effect until 2015, TriNetX automatically converts ICD-9 codes to their corresponding ICD-10 code for diagnoses prior to that date according to general equivalence mappings (GEMS), a guideline on how ICD-9 terminology translates into ICD-10. Individuals with associated genetic syndromes were included in the analysis. Control groups were identified including participants of similar age criteria with no documented history of CL/P who had a healthcare visit during the same timeframe. The rate of documented mental health and behavioral disorders and associated medication prescriptions were compared in the 2 years following documentation of a diagnosis of CL/P (for the experimental cohorts) or a general medical visit (for controls). Separate analyses were conducted for pediatric participants ≤18 and adults >18 years of age. Adults were included in the analysis if they had a documented diagnosis code for CL/P after the age of 18, and mental health disorders in this group were recorded any time within 2 years following this documentation. Mental health and behavioral disorders included depressive episode (includes mild, moderate, and severe major depressive disorder, depressive type psychosis, and other depressive episodes including atypical depressive disorder and premenstrual dysphoric disorder), anxiety disorders (includes generalized anxiety disorder, panic disorder, mixed anxiety disorders, and unspecified anxiety disorders/states), attention-deficit hyperactivity disorders (ADHD) (includes predominantly inattentive type, predominantly hyperactive type, combined type, other type, attention-deficit disorder without hyperactivity, unspecified hyperkinetic syndrome, and hyperkinetic syndrome of childhood) and disruptive disorders (includes confined to family context, childhood and adolescent-onset types, aggressive type, socialized/under-socialized conduct disorders, oppositional defiant disorder, other conduct disorders, mixed disturbance of conduct and emotions, and unspecified disturbance of conduct). Mental health medications included selective serotonin reuptake inhibitors, serotonin norepinephrine reuptake inhibitors, tricyclic antidepressants, monoamine oxidase inhibitors, as well as other antidepressants including trazodone, vilazodone, viloxazine, levomilnacipran, mirtazapine, esketamine, nefazodone, bupropion, fluvoxamine, milnacipran, and maprotiline. Behavioral medications included amphetamine and amphetamine-like stimulants including dextroamphetamine, methamphetamine, lisdexamfetamine, amphetamine, serdexmethylphenidate, dexmethylphenidate, and methylphenidate. Individuals were excluded from analysis if they did not have active records for the duration of the study period. Additional sub-analyses were conducted to compare the same outcomes among individuals categorized as CL, CP, or combined CLP. In order to be included in the analysis of a specific cleft type, individuals needed to have the diagnosis code only for that cleft type and could not have any codes for other cleft types documented. To examine the impact of age, cohorts were further stratified into the following age subgroups: 0–3, 4–6, 7–9, 10–12, 13–15, 16–18, and 18+ years. For these analyses, participants were assigned to an age group if they had a documented CL/P diagnosis code within the specified age range and the rate of mental health disorders was assessed over the following 2 years. This was compared to controls who had a healthcare visit during the same age range. As a result, those with extended follow-up may have been included in the analysis of multiple age cohorts. Three year age intervals (eg, 4–6, 7–9, etc.) were chosen to ensure that each age group contained a sufficient sample size for meaningful statistical comparison. Interpretations regarding the 0–3 year age group are made with caution, as the diagnosis of many mental health disorders is not reliably validated before the age of 4.^20^

To evaluate the impact of CHL or OME on behavioral health within the cleft population, further analyses were performed. Only pediatric individuals with CLP or CP were included in this analysis. People with CL were excluded, as those with CL are not at increased risk of CHL/OME, and adults were excluded as the condition is more commonly diagnosed in children. Individuals with CLP or CP who had documented CHL/OME at any time in the duration of their medical record were compared to those without CHL/OME.

### Statistical Analysis

All statistical analyses were conducted within the TriNetX platform utilizing the “Compare Outcomes Tool,” which is based on the programming languages R, Python, and JAVA. The platform allowed for real-time analyses of specified cohorts with CL/P compared to the control cohorts representative of the general population without CL/P. As this was a retrospective cohort study, relative risks and 95% confidence intervals (CI) were calculated comparing the differences in rates of outcomes specified via ICD-10 codes between experimental groups and controls. To mitigate potential confounding factors, analyses were conducted following 1:1 propensity score matching for age and sex, ensuring that these key demographic variables were balanced between the groups. This technique involves estimating the probability (propensity score) of each participant receiving a particular treatment or exposure based on observed covariates (age and sex). Once these scores are calculated, each participant in the treatment group is matched with a participant in the control group with a similar propensity score. The matching is done on a 1:1 basis, meaning each participant from the treatment group is paired with one participant from the control group with the closest propensity score. This helps ensure that the comparison between groups is as balanced as possible in terms of observed characteristics, thus reducing bias. A 1:1 matching ratio was employed using the nearest neighbor method without replacement, with a caliper width set at 0.1 times the standard deviation to ensure precise matching. Controls were drawn from the same 92 HCOs as the cleft cohort, but matching by geographic region was not feasible due to limitations of the TriNetX platform. In TriNetX, propensity scores are calculated through logistic regression by arranging covariate matrices in a randomized row order. Statistical significance was defined as a *P*-value <.05.

## Results

### Cohort Demographics

**Figure 1.**
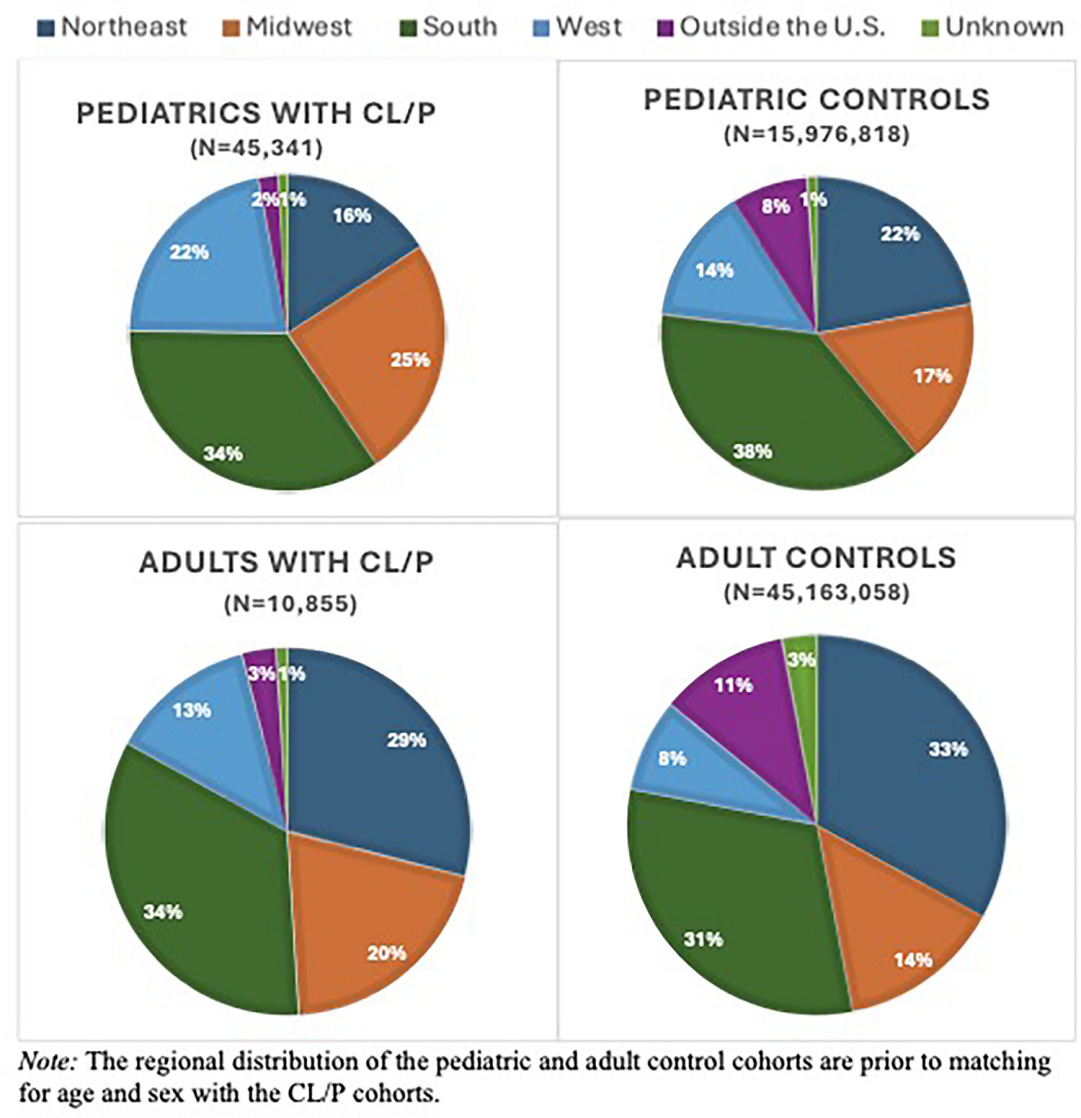
Regional geographic distribution of participants with cleft lip and/or palate and controls.

There were 45,341 pediatric individuals ≤18 years of age identified with a diagnosis of CL/P. Among them were 53.1% males (*n* = 24,063), and the mean age was 5.1 ± 5.3 years. There were 15,976,818 pediatric controls identified with no history of CL/P. Additionally, 10,855 adults >18 years of age with a documented diagnosis of CL/P and 45,163,058 adult controls were identified. On average, adults with CL/P were 36.7 ± 16.6 years old and 62.0% were female (*n* = 6727). The complete demographics of the CL/P cohorts, including race and ethnicity, are presented in Table 1. The majority of individuals in both the pediatric and adult CL/P cohorts had an identified race of white and ethnicity of not Hispanic or Latino. The regional geographic distribution of participants from each cohort prior to matching for age and sex is displayed visually in Figure 1. Pediatric and adult participants with CL/P were more likely to be from the Midwest and West compared to controls, while controls were more likely to be from the Northeast or outside the USA. The cohort demographics after matching for age and sex are presented in Table 2.

**Table 1.** Demographics of Participants with Cleft Lip and/or Palate.

	Pediatric participants ≤ 18 years (*n* = 45,341)	Adult participants > 18 years (*n* = 10,855)
Age (years)	5.1 ± 5.3	36.7 ± 16.6
Sex		
Male	24,063 (53.1%)	3939 (36.3%)
Female	20,448 (45.1%)	6727 (62.0%)
Unknown	830 (1.8%)	189 (1.7%)
Race		
White	28,601 (63.1%)	7734 (71.2%)
Black or African American	3666 (8.1%)	806 (7.4%)
Asian	3050 (6.7%)	466 (4.3%)
Other	3508 (7.7%)	598 (5.5%)
American Indian or Alaska Native	223 (0.5%)	62 (0.6%)
Native Hawaiian or Other Pacific Islander	197 (0.4%)	56 (0.5%)
Unknown race	6096 (13.4%)	1133 (10.4%)
Ethnicity		
Not Hispanic or Latino	31,656 (69.8%)	7884 (72.7%)
Hispanic or Latino	8401 (18.5%)	1448 (13.3%)
Unknown ethnicity	5284 (11.7%)	1523 (14.0%)

**Table 2.** Participants with CL/P and matched controls characteristics.

Pediatric participants ≤ 18 years												
	CL/P total (*n* = 45,341)	Control total (*n* = 45,341)	*P*	CP (*n* = 20,137)	Controls (*n* = 20,137)	*P*	CL (*n* = 5112)	Controls (*n* = 5112)	*P*	CLP (*n* = 19,041)	Controls (*n* = 19,041)	*P*
Age (M ± SD)	5.12 ± 5.28	5.12 ± 5.28	1.0	4.72 ± 4.84	4.72 ± 4.84	1.0	5.49 ± 5.78	5.49 ± 5.78	1.0	5.49 ± 5.5	5.49 ± 5.5	1.0
Sex												
Male	24,063 (53.1%)	24,063 (53.1%)	1.0	9832 (48.8%)	9832 (48.8%)	1.0	2699 (52.8%)	2699 (52.8%)	1.0	10,988 (57.7%)	10,988 (57.7%)	1.0
Female	20,448 (45.1%)	20,448 (45.1%)	1.0	9806 (48.7%)	9806 (48.7%)	1.0	2380 (46.6%)	2380 (46.6%)	1.0	7764 (40.8%)	7764 (40.8%)	1.0
Unknown	830 (1.8%)	830 (1.8%)	1.0	499 (2.5%)	499 (2.5%)	1.0	33 (0.7%)	33 (0.7%)	1.0	289 (1.5%)	289 (1.5%)	1.0
Adult Participants >18 years												
Variable	CL/P total (*n* = 10,855)	Control total (*n* = 10,855)	*P*	CP (*n* = 4440)	Controls (*n* = 4440)	*P*	CL (*n* = 2041)	Controls (*n* = 2041)	*P*	CLP (*n* = 4066)	Controls (*n* = 4066)	*P*
Age (M ± SD)	36.7 ± 16.6	36.7 ± 16.6	1.0	42.7 ± 17.6	42.7 ± 17.6	1.0	36.3 ± 14.5	36.3 ± 14.5	1.0	31.2 ± 14.1	31.2 ± 14.1	1.0
Sex												
Male	3939 (36.3%)	3939 (36.3%)	1.0	1746 (39.3%)	1746 (39.3%)	1.0	478 (23.4%)	478 (23.4%)	1.0	1573 (38.7%)	1573 (38.7%)	1.0
Female	6727 (62.0%)	6727 (62.0%)	1.0	2579 (58.1%)	2579 (58.1%)	1.0	1535 (75.2%)	1535 (75.2%)	1.0	2448 (60.2%)	2448 (60.2%)	1.0
Unknown	189 (1.7%)	189 (1.7%)	1.0	115 (1.7%)	115 (1.7%)	1.0	28 (1.4%)	28 (1.4%)	1.0	45 (1.1%)	45 (1.1%)	1.0

*Note:* It was not possible to determine the specific cleft subtype for 1051 pediatric and 308 adult patients. Therefore, the total group of patients with CL/P is slightly greater than the sum of individual cleft subtypes.

Abbreviations: M, mean; SD, standard deviation.

### Mental Health and Behavioral Disorders in Individuals with CL/P

After matching for age and sex, the pediatric and adult cohorts with CL/P both experienced significantly increased rates of depressive episodes, anxiety disorders, ADHD, and disruptive disorders compared to controls (all *P* < .001; Table 3). They also had significantly increased rates of mental health prescriptions as well as stimulant prescriptions (all *P* < .001; Table 3).

**Table 3.** Diagnoses and Prescriptions in Participants with Cleft Lip and/or Palate Compared to Matched Controls.

Pediatric participants ≤18 years																
	CL/P total (*n* = 45,341)	Control total (*n* = 45,341)	RR (95% CI)	*P*	CP (*n* = 20,137)	Controls (*n* = 20,137)	RR (95% CI)	*P*	CL (*n* = 5112)	Controls (*n* = 5112)	RR (95% CI)	*P*	CLP (*n* = 19,041)	Controls (*n* = 19,041)	RR (95% CI)	*P*
Depressive episode	2386 (5.3%)	444 (1.0%)	5.37 (4.86–5.94)	<.001	1192 (5.9%)	167 (0.8%)	7.14 (6.08–8.38)	<0.001	876 (17.1%)	62 (1.2%)	14.13 (10.95–18.23)	<.001	309 (1.6%)	209 (1.1%)	1.48 (1.24–1.76)	<.001
Anxiety disorders	2799 (6.2%)	704 (1.6%)	3.98 (3.66–4.31)	<.001	1470 (7.3%)	296 (1.5%)	4.97 (4.39–5.62)	<0.001	812 (15.9%)	105 (2.1%)	7.73 (6.33–9.44)	<.001	507 (2.7%)	326 (1.7%)	1.56 (1.36–1.79)	<.001
ADHD	3262 (7.2%)	1039 (2.3%)	3.14 (2.93–3.36)	<.001	1627 (8.1%)	437 (2.2%)	3.72 (3.36–4.13)	<0.001	925 (18.1%)	119 (2.3%)	7.77 (6.45–9.37)	<.001	705 (3.7%)	488 (2.6%)	1.45 (1.29–1.62)	<.001
Disruptive disorders	2834 (6.3%)	518 (1.1%)	5.47 (4.99–6.00)	<.001	1507 (7.5%)	232 (1.2%)	6.50 (5.67–7.45)	<0.001	949 (18.6%)	64 (1.3%)	14.83 (11.55–19.04)	<.001	366 (1.9%)	210 (1.1%)	1.74 (1.47–2.06)	<.001
Mental health prescriptions	1469 (3.2%)	634 (1.40%)	2.32 (2.11–2.54)	<.001	719 (3.6%)	251 (1.2%)	2.87 (2.48–3.30)	<0.001	372 (7.28%)	97 (1.90%)	3.84 (3.08–4.78)	<.001	378 (2.0%)	299 (1.6%)	1.26 (1.09–1.47)	.002
Stimulant prescriptions	1701 (3.8%)	728 (1.6%)	2.34 (2.14–2.55)	<.001	983 (4.9%)	287 (1.4%)	3.43 (3.01–3.90)	<0.001	279 (5.5%)	79 (1.5%)	3.53 (2.76–4.52)	<.001	442 (2.3%)	334 (1.8%)	1.32 (1.15–1.52)	<.001
*Adult participants >18 years*																
	CL/P total (*n* = 10,855)	Control total (*n* = 10,855)	RR (95% CI)	*P*	CP (*n* = 4440)	Controls (*n* = 4440)	RR (95% CI)	*P*	CL (*n* = 2041)	Controls (*n* = 2041)	RR (95% CI)	*P*	CLP (*n* = 4066)	Controls (*n* = 4066)	RR (95% CI)	*P*
Depressive episode	2760 (25.4%)	1408 (13.0%)	1.96 (1.85–2.08)	<.001	1072 (24.1%)	549 (12.4%)	1.95 (1.78–2.15)	<0.001	918 (45.0%)	247 (12.1%)	3.72 (3.28–4.22)	<.001	710 (17.5%)	509 (12.5%)	1.40 (1.26–1.55)	<.001
Anxiety disorders	2870 (26.4%)	1996 (18.4%)	1.44 (1.37–1.51)	<.001	1171 (26.4%)	767 (17.3%)	1.53 (1.41–1.66)	<0.001	894 (43.8%)	383 (18.8%)	2.33 (2.11–2.59)	<.001	722 (17.8%)	741 (18.2%)	0.97 (0.89–1.07)	.58
ADHD	911 (8.4%)	407 (3.7%)	2.24 (2.0–2.51)	<.001	208 (4.7%)	141 (3.2%)	1.48 (1.20–1.82)	<0.001	498 (24.4%)	61 (3.0%)	8.16 (6.30–10.57)	<.001	185 (4.6%)	208 (5.1%)	0.89 (0.73–1.08)	.23
Disruptive disorders	507 (4.7%)	47 (0.4%)	10.79 (8.0–14.5)	<.001	118 (2.7%)	20 (0.5%)	5.90 (3.68–9.46)	<0.001	292 (14.3%)	≤10 (≤0.5%)	–	–	86 (2.1%)	25 (0.6%)	3.44 (2.21–5.36)	<.001
Mental health prescriptions	2824 (26.0%)	2024 (18.6%)	1.40 (1.33–1.47)	<.001	1267 (28.5%)	849 (19.1%)	1.49 (1.38–1.61)	<0.001	755 (37.0%)	385 (18.9%)	1.96 (1.76–2.18)	<.001	734 (18.1%)	737 (18.1%)	1.0 (0.91–1.09)	.93
Stimulant prescriptions	437 (4.0%)	346 (3.2%)	1.26 (1.10–1.45)	<.001	151 (3.4%)	126 (2.8%)	1.20 (0.95–1.51)	0.13	150 (7.3%)	52 (2.5%)	2.89 (2.12–3.93)	<.001	127 (3.1%)	169 (4.2%)	0.75 (0.60–0.94)	.01

*Note:* For outcomes with ≤10 patients, TriNetX rounds to 10. Therefore, statistical analyses could not be calculated for these outcomes.

It was not possible to determine the specific cleft subtype for 1051 pediatric and 308 adult patients. Therefore, the total group of patients with CL/P is slightly greater than the sum of individual cleft subtypes.

Abbreviations: RR, relative risk; CI, confidence interval; ADHD, attention-deficit hyperactivity disorder

### Stratification by Cleft Subtype

There were 44,290 pediatric individuals who had their specific cleft subtype documented, with the remaining 1051 who did not have this documentation excluded from this analysis. Among the adults, 10,547 had a specific documented cleft subtype, with the remaining 308 excluded as above. Among the pediatric individuals, 45.5% (*n* = 20,137) had CP, 11.5% (*n* = 5112) had CL, and 43.0% (*n* = 19,041) had CLP. For the adults, 42.1% (*n* = 4440) had CP, 19.4% (*n* = 2041) had CL, and 38.5% (*n* = 4066) had CLP (Table 3). In both pediatric and adult populations, CP had a significantly higher rate of all mental health and behavioral disorders and prescriptions compared to controls (all *P* < .001), except for no significant difference in stimulant prescriptions among adults (*P* = .13; Table 3). Compared to matched controls, children and adults with CL had the highest rates of mental health and behavioral disorders, as well as prescriptions (all *P* < .001; Table 3). Children with CLP had statistically significant, but the smallest rates of diagnoses and prescriptions compared to controls (all *P* < .002). Adults with CLP had statistically significant higher rates of depressive episodes and disruptive disorders than controls (both *P* < .001); however, there were no differences in anxiety disorders, ADHD, or mental health prescriptions (all *P* > .05). In contrast to the pattern of higher risk associated with CL/P, there was a lower rate of stimulant prescriptions for adults with CLP in comparison to controls (*P* = .01; Table 3).

### Stratification by Age

**Figure 2.**
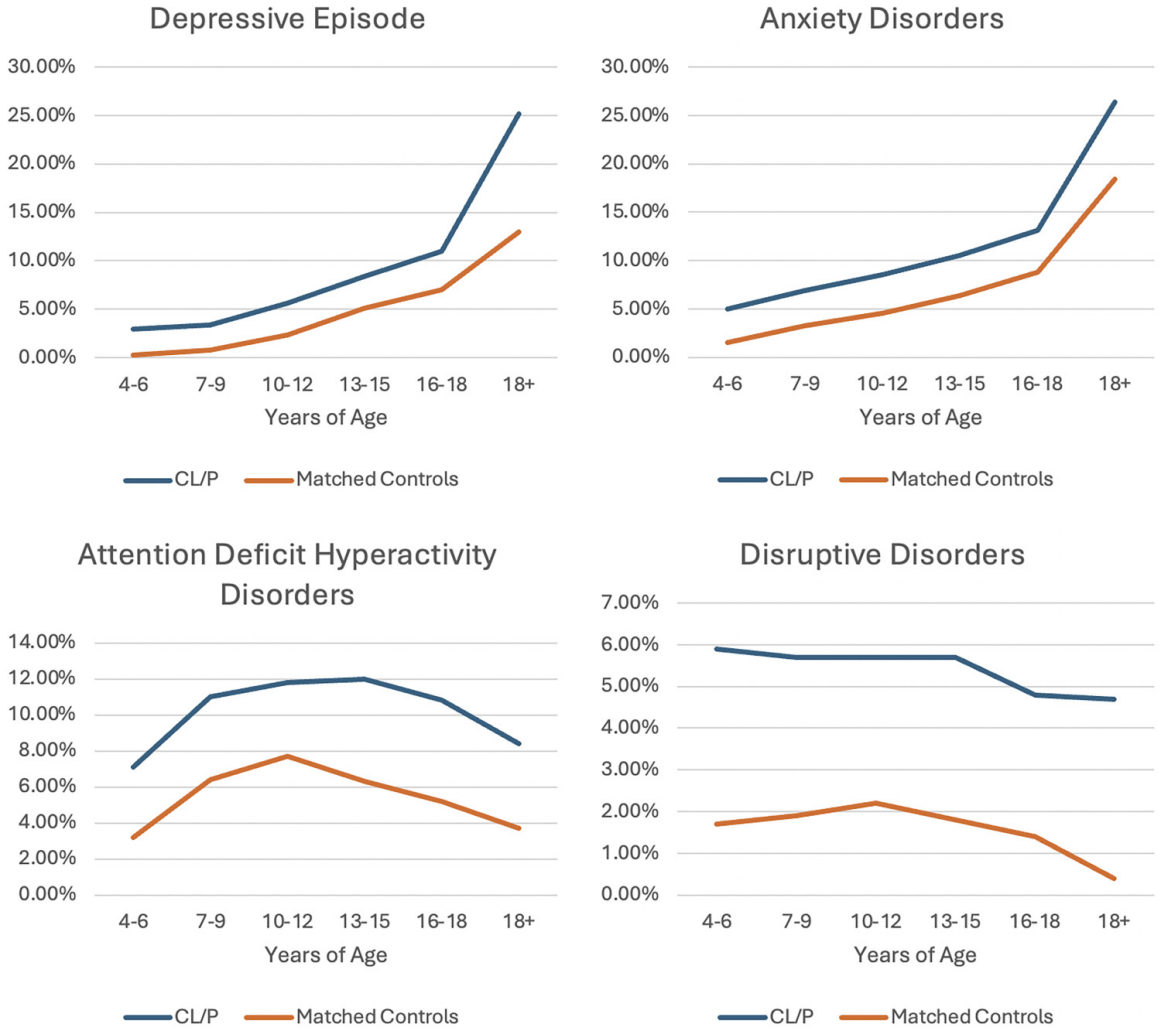
Diagnoses in participants with cleft lip and/or palate compared to matched controls, stratified by age.

**Figure 3.**
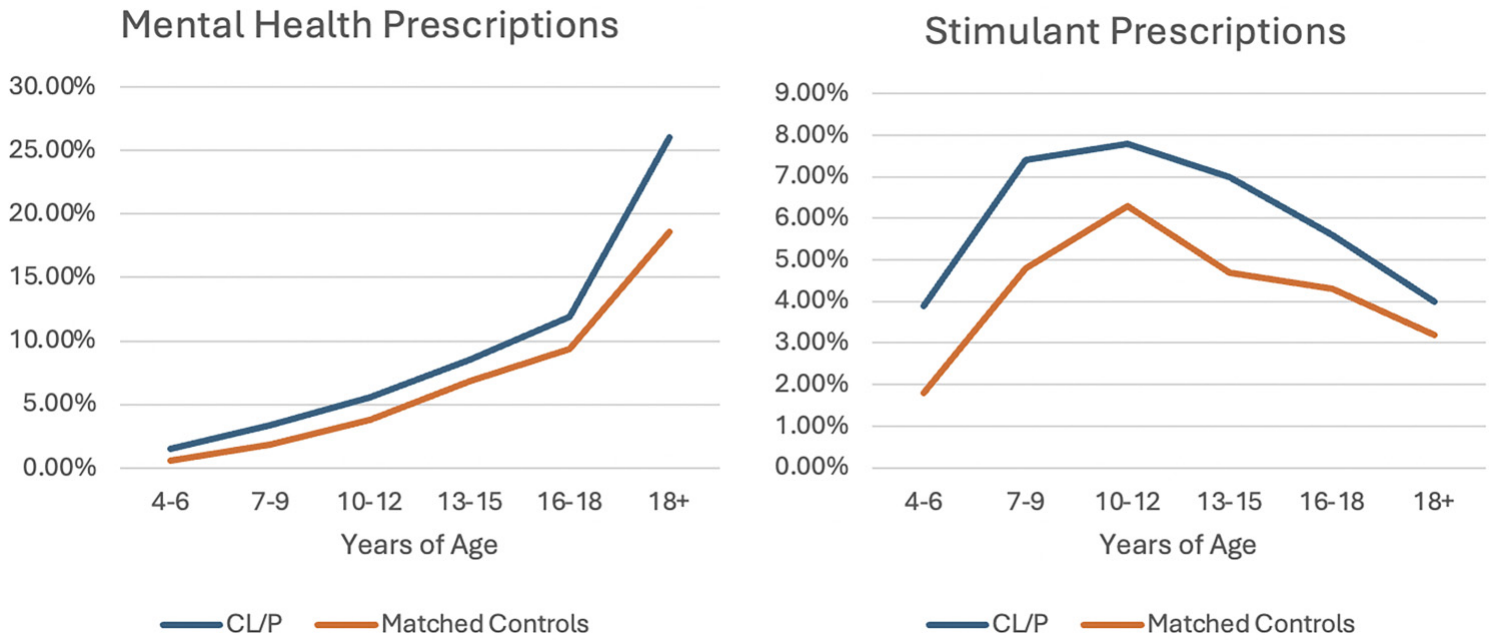
Mental health and behavioral prescriptions in participants with cleft lip and/or palate compared to matched controls, stratified by age.

Individuals with CL/P in all analyzed age categories (0–3, 4–6, 7–9, 10–12, 13–15, 16–18, and 18+ years) experienced significantly higher rates of all outcomes compared to age- and sex-matched controls (all *P* < 0.002; Table 4). Outcome trends by age are displayed graphically in Figures 2 and 3. Outcome patterns for CL/P closely mirrored those of controls. For instance, as age increased, diagnoses of depressive episodes and anxiety disorders, as well as prescriptions for mental health medications, became more prevalent in both CL/P and control groups. Similarly, diagnoses of ADHD and stimulant prescriptions peaked during adolescence, then declined in the teen and adult years for people with and without CL/P. The rate of disruptive disorders remained relatively consistent across age groups.

**Table 4.** Diagnoses and Prescriptions in Participants with Cleft Lip and/or Palate Compared to Matched Controls, Stratified by Age.

	Ages 4–6 years				Ages 7–9 years				Ages 10–12 years			
	CL/P (*n* = 13,619)	Controls (*n* = 13,619)	RR (95% CI)	*P*	CL/P (*n* = 11,537)	Controls (*n* = 11,537)	RR (95% CI)	*P*	CL/P (*n* = 9272)	Controls (*n* = 9272)	RR (95% CI)	*P*
Depressive episode	392 (2.9%)	38 (0.3%)	10.31 (7.40–14.38)	<.001	395 (3.4%)	88 (0.8%)	4.49 (3.57–5.65)	<.001	520 (5.6%)	217 (2.3%)	2.40 (2.05–2.80)	<.001
Anxiety disorders	675 (5.0%)	209 (1.5%)	3.23 (2.77–3.77)	<.001	793 (6.9%)	376 (3.3%)	2.11 (1.87–2.38)	<.001	784 (8.5%)	431 (4.6%)	1.82 (1.62–2.04)	<.001
ADHD	962 (7.1%)	437 (3.20%)	2.20 (1.97–2.46)	<.001	1274 (11.0%)	738 (6.4%)	1.73 (1.58–1.88)	<.001	1091 (11.8%)	712 (7.7%)	1.53 (1.40–1.68)	<.001
Disruptive disorders	800 (5.9%)	234 (1.7%)	3.42 (2.96–3.95)	<.001	662 (5.7%)	224 (1.9%)	2.96 (2.55–3.43)	<.001	526 (5.7%)	200 (2.2%)	2.63 (2.24–3.09)	<.001
Mental health prescriptions	208 (1.5%)	77 (0.6%)	2.70 (2.08–3.51)	<.001	388 (3.4%)	213 (1.8%)	1.82 (1.54–2.15)	<.001	518 (5.6%)	351 (3.8%)	1.48 (1.29–1.69)	<.001
Stimulant prescriptions	533 (3.9%)	247 (1.8%)	2.16 (1.86–2.51)	<.001	859 (7.4%)	552 (4.8%)	1.56 (1.40–1.73)	<.001	722 (7.8%)	580 (6.3%)	1.25 (1.12–1.38)	<.001
	Ages 13–15 years				Ages 16–18 years							
	CL/P (*n* = 7080)	Controls (*n* = 7080)	RR (95% CI)	*P*	CL/P (*n* = 5125)	Controls (*n* = 5125)	RR (95% CI)	*P*				
Depressive episode	598 (8.4%)	362 (5.1%)	1.65 (1.46–1.87)	<.001	562 (11.0%)	358 (7.0%)	1.57 (1.38–1.78)	<.001				
Anxiety disorders	741 (10.5%)	453 (6.4%)	1.64 (1.46–1.83)	<.001	673 (13.1%)	451 (8.8%)	1.49 (1.33–1.67)	<.001				
ADHD	850 (12.0%)	444 (6.3%)	1.91 (1.72–2.14)	<.001	551 (10.8%)	267 (5.2%)	2.06 (1.79–2.38)	<.001				
Disruptive disorders	400 (5.7%)	129 (1.8%)	3.10 (2.55–3.77)	<.001	245 (4.8%)	70 (1.4%)	3.50 (2.69–4.55)	<.001				
Mental health prescriptions	599 (8.5%)	484 (6.8%)	1.24 (1.10–1.39)	<.001	610 (11.9%)	483 (9.4%)	1.26 (1.13–1.41)	<.001				
Stimulant prescriptions	495 (7.0%)	336 (4.7%)	1.47 (1.29–1.69)	<.001	287 (5.6%)	220 (4.3%)	1.31 (1.10–1.55)	.002				

*Note:* The total number of patients adds to greater than total cohort because some patients had prolonged follow up and therefore were able to be included in the analyses of multiple age groups.

Abbreviations: RR, relative risk; CI, confidence interval; ADHD, attention-deficit hyperactivity disorder.

Mental health outcomes for the 0–3 year age group were not included in the tables or figures, as these diagnoses are not typically validated before the age of 4. However, children aged 0–3 with CL/P exhibited significantly higher rates of all mental health diagnoses and prescriptions compared to those without CL/P of similar age (all *P* < .001). Within the 0–3 year old CL/P group, the rates of mental health outcomes ranged from 0.4% for stimulant prescriptions to 4.1% for disruptive disorders. In contrast, the 0–3 year non-CL/P group showed outcome rates ranging from 0.1% for stimulant prescriptions to 0.7% for disruptive disorders. Overall, children ages 0–3 years with CL/P were diagnosed with mental health conditions at rates 5–9 times higher than their peers without CL/P, and were prescribed medications at 3–4 times the rate.

### Effect of CHL

There were 62.2% (*n* = 24,375) of pediatric individuals with CP or CLP who had an associated diagnosis of CHL or OME. Compared to individuals with cleft without these associated diagnoses, those with CHL/OME experienced significantly increased rates of all mental and behavioral diagnoses and prescriptions (all *P* < 0.001; Table 5).

**Table 5.** Diagnoses and Prescriptions in Pediatric Participants with Cleft Palate with or without Cleft Lip with and without Documented Otitis Media with Effusion or Conductive Hearing Loss.

	CP ± L with CHL/OME (*n* = 24,375; 13,317 after matching)	CP ± L without CHL/OME (*n* = 14,803; 13,317 after matching)	RR (95% CI)	*P*
Depressive episode	695 (5.2%)	346 (2.6%)	2.01 (1.77–2.28)	<.001
Anxiety disorders	931 (7.0%)	519 (3.9%)	1.79 (1.62–1.99)	<.001
ADHD	1135 (8.5%)	684 (5.1%)	1.66 (1.51–1.82)	<.001
Disruptive disorders	862 (6.5%)	422 (3.2%)	2.04 (1.82–2.29)	<.001
Mental health prescriptions	530 (4.0%)	354 (2.7%)	1.50 (1.31–1.71)	<.001
Stimulant prescriptions	720 (5.4%)	467 (3.5%)	1.54 (1.38–1.73)	<.001

Abbreviations: RR, relative risk; CI, confidence interval; ADHD, attention-deficit hyperactivity disorder; CHL, conductive hearing loss; OME, otitis media with effusion.

## Discussion

Our current study confirms the increased risk of mental health and behavioral disorders associated with CL/P,^9‐13^ while providing additional data into how mental health outcomes vary across cleft subtypes. Consistent with prior literature, we found significantly higher rates of mental health conditions among individuals with CL/P compared to matched controls without clefts. One study of 1158 children with CL/P in Taiwan found a higher likelihood of developing schizophrenia, ADHD, and autism spectrum disorder, though no association was found between CL/P and major depressive disorder.^9^ A large Danish cohort study reported that individuals with oral clefts had a higher risk of psychiatric diagnoses compared to those without, particularly among those with CP or CLP, while those with CL showed no significant increase in risk.^10^ Similarly, a Swedish cohort study found elevated psychiatric and neurodevelopmental risks across all cleft types, with children with CP experiencing the highest increases in risk, including for psychotic disorders, ADHD, and behavioral and emotional disorders.^11^ A Danish study investigated psychotropic medication use and mental health visits and found that individuals with CP or CLP had a higher risk of using antipsychotics and stimulants, along with increased psychiatrist visits for CP.^12^ A Swedish population-based study also found that adolescents with CL or CP were more likely to use psychotropic drugs compared to those without oral clefts; however, individuals with CLP did not show a significant increase in psychotropic drug use.^13^ However, contrary to some of the prior research which has reported that individuals with CP or CLP may have the highest rate of psychiatric comorbidities,^10‐12^ in the current study those with CL exhibited the most pronounced increases in these conditions, followed by CP, while those with combined CLP showed comparatively smaller or no increases.

Children with CL/P often face significant challenges related to speech and language development,^21^ which can hinder their ability to communicate effectively and engage in social interactions.^22^ These difficulties may lead to increased feelings of isolation, frustration, and low self-esteem.^23^ Additionally, the visible nature of CL/P can result in social stigmatization and bullying,^24^ further exacerbating psychological distress. One study reported that 69% of individuals with CL/P are victims of bullying in school.^25^ The ongoing need for multiple surgeries, medical treatments, and years of follow-up can contribute to emotional strain,^26^ while associated conditions like CHL, OME, and speech difficulty may compound these issues. Together, these factors create a complex interplay that heightens the risk of mental health disorders in this population. Notably, the data from this study indicated that the higher mental health impacts associated with CL/P were present across age groups early childhood to adulthood, highlighting the long-term psychological burden faced by these individuals. Notably, individuals with CL/P consistently exhibited higher rates across all categories compared to those without CL/P; however, the overall trends were similar across time between age groups. Rates of depressive episodes, anxiety disorders, and mental health medication use increased with age, while ADHD and stimulant medication use peaked during adolescence, and disruptive disorders plateaued across age groups. The finding that children aged 0–3 with CL/P were diagnosed with mental health disorders and prescribed medications at 5–9 and 3–4 times the rate of their peers, respectively, despite the lack of validated diagnostic criteria for mental health disorders in this age group, raises concerns about the accuracy of these early diagnoses. This suggests that the higher rates may reflect potential over diagnosis, bias by health professionals, or greater treatment seeking or interaction with healthcare systems by families of children with CL/P. It highlights the need for further research to better understand the validity of mental health diagnoses in this age group and to ensure that early interventions are based on reliable, evidence-based evaluations.

Although there are effective surgical management options for CL/P, one study showed that Swedish adults who received treatment as children prior to the 1980s reported significant dissatisfaction with facial appearance and nearly half wished to have further operations.^27^ However, existing evidence suggests that the relationship between physical appearance and psychological well-being is more complex and nuanced. In fact, Moss demonstrated that differences in objective appearance contribute only minimally to variations in an individual's appearance-related well-being or self-consciousness.^28^ Rather, subjective factors, such as individual coping strategies and social support, play a significant role in determining body image and psychological impact. This highlights the importance of addressing not only the physical aspects of the condition but also the psychological and emotional factors that may have an even greater effect on overall well-being.

It is not clear why individuals with CL in this study had high rates of mental health diagnoses and prescriptions, which is generally not consistent with prior studies and requires further investigation and replication across populations. A possible contributing factor to the difference in mental health outcomes by cleft type could be that children with CL may be less likely to be followed by a cleft team, while children with CP and CLP may have received ongoing psychological support through their cleft team which could have mitigated some of their psychological concerns. This suggests that this CL subgroup may be particularly vulnerable and may benefit from targeted early psychological evaluation and intervention to mitigate the long-term impact of these mental health challenges.

Those with CLP exhibited the smallest elevated rates of disorders and prescriptions, including those with CP. Although many outcomes in the CLP group were still statistically significantly higher compared to a matched non-cleft population, it is unclear why those who often require the most cleft care had a lower mental health burden. There is growing evidence that social support and adaptive coping, such as benefit finding, in response to medical stressors may build resiliency with overall similar outcomes to those with a cleft.^29‐31^ Children with CP are also at higher risk for syndromes and learning disorders,^32^ which may be a contributing factor for their increased rate of mental health concerns compared to CLP. Differences across existing studies could be explained by variations in cultural and environmental factors that influence the development of mental health conditions, or by differences in healthcare systems and diagnostic practices between the US and international countries.

Children with CP or CLP and associated CHL/OME experienced notably higher rates of mental health disorders and related prescriptions compared to their peers without these conditions. Hearing impairments place additional strains on communication, social interaction, and academic performance.^33,34^ Research indicates that even unilateral hearing loss can significantly impact academic performance, and that 40% of affected individuals may struggle to engage in regular activities.^35^ These difficulties can amplify feelings of isolation and frustration, exacerbating the psychological distress associated with CL/P. This is most relevant for younger individuals, as the incidence of OME in those with CP is most prevalent at 4–6 years of age.^36^

The robust nature of the TriNetX database suggests the findings are reliable and generalizable; however, several limitations should be considered. First, the study relies on retrospective data dependent on the quality of data entry into electronic medical records. Coding inaccuracies may have led to misclassifications and inaccuracies in terms of the link between the type of cleft pathology and mental health disorders. There is a lack of cleft treatment data in this study. While the conversion of ICD-9 to ICD-10 for diagnoses prior to 2015 is a strength of the database, the GEMS mapping is not exact and may have led to some discrepancies and included a varying range of mental health diagnoses. While propensity score matching was employed to control for age and sex, residual confounding may still exist, particularly in unmeasured socioeconomic factors, environmental influences, or medical comorbidities and syndromes, which are known to impact mental health. TriNetX does not provide important information regarding socioeconomic status such as educational attainment and mean household income, and it was not possible to match based on geographic region. Additionally, while the control cohorts were large and well-balanced, TriNetX only allows for matching utilizing 1:1 propensity score matching. Using larger matched control groups could have further strengthened the study's validity had it been possible. It should be noted that there was a large overrepresentation of females within the pediatric and adult CL/P samples, especially among adults with CL only. This may have biased reported outcomes, and may be an indication that males with CL/P may not be as likely to access care as adults. Regarding medications, it was not possible to determine more detailed information such as dose and number of refills. Further research should also analyze other classes of medications such as non-stimulant medications for ADHD and benzodiazepines for anxiety. Excluding these medications from our analysis may have led to an underestimation of the total number of individuals receiving pharmacologic treatment, as it has been reported that as many as 13.6% of children with ADHD are taking non-stimulant medications such as atomoxetine.^37^ Furthermore, the number of individuals receiving psychotherapy could not be ascertained, likely underestimating the total number treated for mental health conditions.

Despite limitations, overall these findings underscore the importance of a holistic approach to the care of individuals with CL/P, not only addressing their physical and developmental needs but also closely monitoring and managing their mental health. The increased risk of mental health disorders in this population suggests that early intervention and support are critical.^23^

## Conclusion

There is a significant mental health burden faced by pediatric and adult individuals with CL/P, particularly those with CL or associated hearing conditions. These insights should encourage a multidisciplinary approach to care that includes mental health screening and intervention as integral components of treatment for CL/P.

## Supplemental Material

sj-docx-1-cpc-10.1177_10556656251331329 - Supplemental material for Cleft Lip and/or Palate Is Associated with an Increased Prevalence of Mental Health and Behavioral Disorders Supplemental material, sj-docx-1-cpc-10.1177_10556656251331329 for Cleft Lip and/or Palate Is Associated with an Increased Prevalence of Mental Health and Behavioral Disorders by F. Jeffrey Lorenz, Cheng Ma, Aniruddha C. Parikh and Jessyka G. Lighthall in The Cleft Palate Craniofacial Journal
