# Supplementary material for: Cleft Lip and/or Palate Is Associated with an Increased Prevalence of Mental Health and Behavioral Disorders
Source: Cleft Palate Craniofac J. 2025 Apr 30;63(5):1285–96. doi: 10.1177/10556656251331329 (PMC12700003; doi:10.1177/10556656251331329)
Supplement: sj-docx-1-cpc-10.1177_10556656251331329 - Supplemental material for Cleft Lip and/or Palate Is Associated with an Increased Prevalence of Mental Health and Behavioral Disorders [file sj-docx-1-cpc-10.1177_10556656251331329.docx]

**Supplement 1:** Diagnosis (ICD) and Procedure (CPT) Codes

ICD-10 codes are listed first, followed by ICD-9 codes, which were automatically converted to their corresponding ICD-10 codes for diagnoses recorded prior to 2015.

Cleft lip and/or palate:

Q35 Cleft palate (includes Q35.1, Q35.3, Q35.5, Q35.7, Q35.9) (ICD-9 749.0)

Q36 Cleft lip (includes Q36.0, Q36.1, Q36.9) (ICD-9 749.1)

Q37 Cleft palate with cleft lip (includes Q37.0, Q37.1, Q37.2, Q37.3, Q37.4, Q37.5, Q37.8, Q37.9) (ICD-9 749.2)

Mental health disorders:

F32 Depressive episode (includes F32.0, F32.1, F32.2, F32.3, F32.4, F32.5, F32.8, F32.9, F32.A) (ICD-9 296.2, 296.20, 296.21, 296.22, 296.23, 296.24, 296.25, 296.26, 296.82, 298.0, 311)

F41 Anxiety disorders (includes F41.0, F41.1, F41.3, F41.8, F41.9) (ICD-9 300.0, 300.00, 300.01, 300.02, 300.09)

Behavioral disorders:

F90 Attention deficit hyperactivity disorders (includes F90.0, F90.1, F90.2, F90.8, F90.9) (ICD-9 314, 314.0, 314.00, 314.01, 314.1, 314.2, 314.8, 314.9)

F91 Disruptive disorders (includes F91.0, F91.1, F91.2, F91.3, F91.8, F91.9) (ICD-9 312, 312.00, 312.01, 312.02, 312.03, 312.1, 312.10, 312.11, 312.12, 312.13, 312.2, 312.20, 312.21, 312.22, 312.23, 312.4, 312.8, 312.81, 312.82, 312.89, 312.9, 313.81)

Tricyclic antidepressants:

10834 Trimipramine

2597 Clomipramine

3247 Desipramine

3638 Doxepin

5691 Imipramine

704 Amitriptyline

722 Amoxapine

7531 Nortriptyline

8886 Protriptyline

Monoamine oxidase inhibitor antidepressants:

10734 Tranylcypromine

6011 Isocarboxazid

8123 Phenelzine

9639 Selegiline

Antidepressants, other:

10737 Trazodone

1086769 Vilazodone

11196 Viloxazine

15996 Mirtazapine

2119365 Esketamine

2556 Citalopram

31565 Nefazodone

321988 Escitalopram

32937 Paroxetine

36437 Sertraline

39786 Venlafaxine

42347 Bupropion

42355 Fluvoxamine

4493 Fluoxetine

588250 Milnacipran

6646 Maprotiline

72625 Duloxetine

734064 Desvenlafaxine

Amphetamines/Amphetamine-like stimulants:

3288 Dextroamphetamine

6816 Methamphetamine

700810 Lisdexamfetamine

725 Amphetamine

2562176 Serdexmethylphenidate

352372 Dexmethylphenidate

6901 Methylphenidate
